# Involvement of Endogenous Retroviruses in Prion Diseases

**DOI:** 10.3390/pathogens2030533

**Published:** 2013-08-12

**Authors:** Yun-Jung Lee, Byung-Hoon Jeong, Eun-Kyung Choi, Yong-Sun Kim

**Affiliations:** 1 Ilsong Institute of Life Science, Hallym University, Anyang, Gyeonggi-do 431-060, Republic of Korea; E-mails: jjung0301@hallym.ac.kr (Y.J.L.); bhjeong@hallym.ac.kr (B.H.J.); ekchoi@hallym.ac.kr (E.K.C.); 2 Department of Microbiology, College of Medicine, Hallym University, Chuncheon, Gangwon-do 200-702, Republic of Korea

**Keywords:** prion diseases, Creutzfeldt-Jakob disease (CJD), endogenous retroviruses (ERVs)

## Abstract

For millions of years, vertebrates have been continuously exposed to infection by retroviruses. Ancient retroviral infection of germline cells resulted in the formation and accumulation of inherited retrovirus sequences in host genomes. These inherited retroviruses are referred to as endogenous retroviruses (ERVs), and recent estimates have revealed that a significant portion of animal genomes is made up of ERVs. Although various host factors have suppressed ERV activation, both positive and negative functions have been reported for some ERVs in normal and abnormal physiological conditions, such as in disease states. Similar to other complex diseases, ERV activation has been observed in prion diseases, and this review will discuss the potential involvement of ERVs in prion diseases.

## 1. Prion Diseases

Prion diseases, also known as transmissible spongiform encephalopathies (TSEs), are a group of invariably fatal neurodegenerative disorders of the central nervous system (CNS). Prion diseases affect a broad spectrum of mammals and have different names depending on the host species, including scrapie in sheep, bovine spongiform encephalopathy (BSE) in cattle and Creutzfeldt-Jakob disease (CJD) in humans. Because the infectious agent responsible for prion disease can be spread between animals, prion diseases have been regarded as a serious public health risk. However, despite its virus-like properties, the prion infectious agent is resistant to virus-inactivating procedures and is devoid of nucleic acid. At present, unconventional proteinaceous infectious particles (called prions) are known to be the causative agent of prion diseases, with many studies supporting this protein-only hypothesis since it was suggested by Stanley B. Prusiner in 1982. Prions are composed of a disease-associated abnormal isoform (denoted PrP^Sc^ for scrapie, the first well-characterized prion disease) that is formed by a conversion process from normal cell-surface glycoprotein (termed PrP^C^ for cellular). PrP^Sc^ acquires characteristic properties resulting from this conformational change, such as a β-sheet enriched structure and partial protease resistance, whereas endogenous PrP^C^ contains a predominantly α-helical structure and is easily degraded by proteases. PrP^Sc^ is found in most prion diseases and is used as a common biochemical hallmark [1,2].

In humans, prion diseases can be divided etiologically into sporadic, genetic and acquired forms by infection of prion-contaminated agents. Most of the human prion diseases are classified as CJD; approximately 85% of all cases of CJD occur sporadically, whereas 10-15% are caused by mutations in the prion protein gene (*PRNP*). More than 50 different autosomal dominant pathogenic *PRNP* mutations have been reported to cause inheritable CJD. In addition, fewer than 1% of cases are acquired iatrogenically following treatment with contaminated growth hormone or in recipients receiving human dura mater or corneal transplants. In common with other prion diseases, the central features of CJD are the deposition of PrP^Sc^ amyloid plaques, spongiform vacuolation, astrocytic proliferation and neuronal cell loss [1,3]. 

PrP^C^ was identified as the key substrate that is converted to PrP^Sc^ in studies showing that the typical neurological dysfunction associated with prion disease did not develop when the *PRNP* was ablated and that prion disease could be generated by inoculation with recombinant PrP (rPrP) fibrils prepared *in vitro* [4,5,6]. However, the spontaneous transition mechanism for the host encoding of PrP^C^ into PrP^Sc^ has not been clearly elucidated. Under physiological conditions, the presence of a cellular factor for prion formation in the host cannot be entirely ruled out. Because biological macromolecules, including sulfated glycosaminoglycan and specific nucleic acids, can not only interact with PrP^C^ but can also lead to efficient proteinase-resistant PrP (PrP^res^) formation *in vitro*, several molecules have been considered as candidate catalysts for PrP conversion in the host [7,8,9,10,11]. These possible interaction-partners of PrP^C^ have been called protein X and, furthermore, PrP^C^ contains discontinuous structure, the loop (residue 165–171) and carboxy-terminus of helix C, to which protein X could bind [12]. 

## 2. Endogenous Retroviruses

All genomes in the vertebrate class include chromosomally integrated copies of retroviral sequences that are referred to as endogenous retroviruses (ERVs). Because these ERV sequences have entered the host genome through spontaneous retroviral infection of germ line cells, their sequences and structures are highly analogous to those of exogenous retroviruses. However, ERVs can be identified by their vertical inheritance from one generation to the next according to Mendelian genetics, which distinguishes them from exogenous retroviruses. With the completion of the genome project, a substantial portion of the mammalian genome was identified as "junk DNA", including retrotransposons, which can self-amplify *via* an RNA intermediate. In addition, the long terminal repeat (LTR)-containing group of retroelements has been classified as ERVs, and a significant part of the animal genome is made up of ERVs. For example, recent estimates have revealed that the human and mouse genomes contain approximately 8% and 10% ERVs, respectively [13,14,15]. 

ERVs retain some hallmarks of exogenous retroviruses in their sequence composition and genome structure, including *gag*, *pol*, *pro* and *env* sequences flanked by a LTR that act as an alternate promoter and enhancer. ERVs have been broadly grouped into three distinct classes based on their sequence similarity to exogenous retrovirus genera: ERVs related to the gammaretrovirus genera are termed class I, those related to the betaretrovirus genera are termed class II, and those most related to the spumavirus genera are termed class III. In humans, there are at least 26 phylogenetically distinct retroviral lineages in the genome, as shown by calculating the members of monophyletic groups of human ERVs (HERVs) within retroviral phylogenies; these known HERV families have been also divided into three classes [13,16,17]. Mouse ERVs constitute approximately 0.7% class I, 3% class II and 5.4% class III, and a number of active ERVs, such as the murine leukemia viruses (MuLVs) and mouse mammary tumor virus (MMTV), are distributed across these different classes [13,15,18]. 

ERV sequences are scattered in the host chromosome, with copy numbers ranging from one to thousands as the colonization event is followed by further proviral insertions and retrotranspositions [13,19]. Although ERVs are abundant in the host genome, they are inactive or functionally defective. The initial stage of endogenization, or the formation of proviruses, is inhibited by host defenses against viral infection. Furthermore, to protect against the detrimental effects of ERVs, many host factors have evolved to act at various steps of the retroviral like cycle [20,21]. For example, ERV expression is suppressed *via* a biochemical process that mediates DNA silencing; because mammalian ERVs contain CpG dinucleotides and 5-methyl cytosines, the methylation state of genomic DNA is maintained [22,23]. In addition, the expression of a variety of retroviruses, including ERVs, is restricted by host proteins such as APOBEC3G and TRIM5α; however, the precise mechanism responsible for their effect on ERVs remains to be elucidated [24,25,26,27,28]. Consequently, many proviral sequences are negatively selected and carry a large number of mutations. Otherwise, they could be partially excised from the genome by a recombinational deletion process [29]. 

Although a large percentage of ERVs are replication-defective or are suppressed by host defense mechanisms, some ERVs can be expressed and replicated. The activation of an ERV gene can bring positive co-opted functions to the host, which may be evolutionarily maintained [13,20]. For instance, some ERV proteins may provide important functions during normal development; for example, human protein syncytine-1, which is an ERV Env protein, is involved in the formation of the syncytial layer in the placenta [30,31]. Moreover, the expression of Env proteins encoded by ERVs has been suggested to mediate host resistance to exogenous pathogens [32,33]. However, because of their analogy to exogenous retroviruses, the activation of certain ERVs has been frequently implicated in disease.

## 3. Endogenous Retroviruses and Diseases

Exogenous retroviruses elicit a range of diseases such as cancer, AIDS, autoimmunity and diseases of the CNS [34,35]. The clear similarities between ERVs and exogenous infectious retroviruses have provoked many investigators to study potential relations between ERVs and certain diseases. Although a certain direct role for ERVs in the pathogenesis of clinical diseases should be proved more, specific ERV expression has been reported and has emerged as a possible etiological factor in complex diseases [36,37,38]. 

In the mouse, a number of ERVs are still active, and those with infectious counterparts, such as MuLV and MMTV, have been notably studied for decades. Several lines of evidence show that, in common with their exogenous variants, endogenous MuLV and MMTV are responsible for the leukemias that develop in inbred AKR and GR mice, respectively [13]. In addition, recent studies have suggested that two ERVs isolated from murine melanomas and neuroblastomas, named melanoma-associated retrovirus (MelARV) and Neuro-2a-associated retrovirus, respectively, may promote tumorigenesis. For example, one report showed that MelARV insertions could alter the expression profile of particular genes implicated in metastatic spread. Furthermore, transposition of the intracisternal A particle element, a noninfectious ERV sequence, could induce by insertional mutation in plasmacytoma and myeloma cell lines, and was found to affect oncogenes and cytokine genes [39,40,41]. 

HERVs are more defective than the ERVs of other species, and replication-complete or infectious retroviruses that originate from human endogenous genes have not yet been described. However, a limited number of HERV elements retain their transcriptional activity [13,14]. Indeed, the electron microscopy images of human placentas have revealed retrovirus-like particles budding from the basal membranes of syncytiotrophoblasts [42]. Furthermore, the expression of HERV transcripts or proteins and the observation of retroviral-like particles have been used to associate HERVs with specific diseases. Similar to their mouse counterparts, the etiological roles of HERVs have been implicated in various types of human cancers or tumor cell lines [38]. The detection viral particles in a malignant melanoma cell culture [43,44] and the overexpression of viral proteins encoded by HERV-K in germ cell tumors in several studies may reflect the oncogenic properties of certain HERVs [45,46,47,48]. Furthermore, previous studies have suggested the involvement of cell fusion that is characteristic of syncytin-1 in breast cancer and endometrial carcinoma development [49,50]. In multiple sclerosis, a chronic inflammatory disease of the CNS in which the myelin sheath is damaged, the up-regulation of some HERVs and syncytin-1 protein was observed in patients’ brains, and the abnormal expression of syncytin-1 in astrocytes resulted in the induction of cytokines and reactive oxygen species, with ensuing oligodendrocyte damage [51]. Up-regulation of certain HERV sequences was also detected in patients with neuropsychiatric disorders of unknown etiology, such as schizophrenia and bipolar disorder, in which a significant loss of brain volume had occurred [52,53]. 

Some possible mechanisms by which ERVs could contribute to the development of such diseases are outlined below. First, the retroviral provirus contains enhancer and promoter sequences in LTRs used to process its own RNA, and these elements can influence the transcription of genes adjacent to the insertion site. Alternatively, mutagenesis resulting from the integration of ERVs could lead to abnormal gene regulation in which the transcription of a particular gene may be disrupted. Moreover, the viral proteins encoded by ERVs may be expected to act as etiological factors for disease; for example, ERV-encoded proteins may abrogate repression of oncogenic transcription factor, induce cell fusion or act like 'foreign' antigens. However, further investigation is required to explain the correlations between ERVs and the pathology of these diseases in humans and animals [38,54]. 

## 4. Endogenous Retroviruses in Prion Diseases

As mentioned above, endogenous retroviral elements have been postulated to be involved in various complex diseases including neurological disorders; interestingly, the activation of ERVs has also been observed in prion diseases. First, infection of a senescence-accelerated mouse strain (SAMP8) that develops active ecotropic MuLV with scrapie led to an increase in the MuLV titer [55,56,57] , and the extent of this increase was dependent on the scrapie strain used, suggesting that the informational molecule of different scrapie strains interacts differentially with replication of the retrovirus [58]. In another study, it was shown that strains with high titers of MuLV in the brain, such as SAMP8 and AKR, had shorter incubation periods than a strain without brain MuLV, *i.e*., the senescence-resistant mouse strain (SAMR1). Furthermore, there is an inverse relationship between the level of MuLV expression and the length of the scrapie incubation period in three different strains, among which AKR mice demonstrated the shortest incubation period [59]. This prompted investigators to examine the relationship between scrapie disease and the activation of ERV replication. Infectious MuLVs are encoded by ERVs in mice and can be classified into three groups according their host range: ecotropic viruses, xenotropic viruses and polytropic viruses. The up-regulated transcription of endogenous MuLVs in all three groups was observed in scrapie-infected SAM mice, and these findings confirm and extend the hypothesis that TSEs may be involved in retrovirus replication, as suggested previously. Moreover, in scrapie-infected SAMP8 mice, the immunoreactivity of MuLV was increased in astrocytes that also exhibited PrP^Sc^ immunoreactivity, and the increases in MuLV expression and PrP^Sc^ staining in the same cell types indicated that either synergistic interaction may have occurred and/or the potential for intracellular interaction may have been augmented [60]. 

To evaluate the possible relationship between HERVs and human prion disease, we examined the retroviral sequences in cerebrospinal fluid (CSF) obtained from individuals with sporadic CJD. The frequencies of several HERV families, including HERV-W, HERV-L, FRD and ERV-9, were significantly increased in the CSF of individuals with sporadic CJD compared to the frequencies observed in normal control CSF. In addition, when compared to individuals with other neurodegenerative diseases that exhibit similar symptoms to CJD, such as dementia, the incidence rate of HERV-W and HERV-L were significantly higher in the CSF of sporadic CJD patients. Moreover, the frequency of increased HERV-W and HERV-L in the same samples was much higher in sporadic CJD than in either normal or other neurodegenerative diseases CSF samples, and there was no correlation between individual parameters, such as sex and age [61]. In accordance with our findings, the up-regulation of endogenous gammaretroviruses, including MacERV-4, ERV-9 and HERV-E, was detected in the brains of BSE-infected non-human primates. In particular, the alteration of ERV-9 was observed in both macaque-adapted BSE and sporadic CJD models. These data, which confirm the activation of ERVs in a primate prion model, support the concept that there may be an interaction between prion pathogenesis and the components of ERVs [62]. 

Similar to other pathogenic conditions, such as autoimmunity and other neurological disorders, the crucial evidence required to explain the role of ERVs in the pathogenesis of prion disease remains elusive. Nevertheless, it is possible that ERVs that are activated as a consequence of altered physiological conditions may contribute to the development of prion diseases, albeit by currently unknown mechanisms. Potentially, components of ERVs may act as cofactors to enhance prion conversion, and this hypothesis is supported by the previous finding that retroviral sequences co-sediment with infectivity in scrapie. For example, small, highly structured RNAs were shown to have the capacity to participate in the conversion of human recombinant PrP^Sen^ to PrP^res^ [63], and RNA molecules were shown to stimulate prion protein conversion [11,64]. In addition, a recent study showed that co-infection of small-ruminant lentiviruses and PrP^Sc^ significantly enhanced PrP^Sc^ accumulation within cells [65]. Furthermore, replicate-competent or -incompetent ERV particles directly correlate with prion infectivity, as previous studies have shown that molony MuLV (MoMuLV), which belongs to ecotropic MuLV, strongly enhances the release of scrapie infectivity and that MoMuLV virions were specifically immunoprecipitated by anti-PrP antibodies [66,67]. Alternatively, ERV transcripts and the potential expression of their proteins may be involved in the progression of CJD and other prion diseases. Previously, a number of studies demonstrated that the RNA expression of ERVs and ERV-encoded proteins was associated with human diseases. According to these studies, ERV proteins may be closely implicated in the pathogenesis of inflammatory diseases, such as multiple sclerosis and rheumatoid arthritis (RA) [51,54,68]. In prion diseases, a correlation between the inflammatory process and neurodegeneration has been suggested because of the activation of large numbers of glial cells and the up-regulated expression of proinflammatory cytokines, which are pathological features of prion diseases [69,70]. 

Finally, previous finding was showed a significant increase in the individual or combination detection rate of W and L type of HERVs in sporadic CJD CSF compared to control and other neurodegenerative diseases CSF [61]. In addition, HERV-L has not been correlated with any other diseases in which cancer, autoimmune diseases and multiple sclerosis are included. These findings suggest that certain HERVs in the CSF may be of special importance in sporadic CJD and might be considered as a candidate for biomarker of sporadic CJD during clinical illness. Although other types of HERV family should be screen in the CSF of sporadic CJD, some HERVs could be used in conjunction with other markers, such as 14-3-3 and tau proteins, to improve the sensitivity of diagnosis.

## 5. Conclusions

Retroviral elements can be transcribed in the CNS of humans and animals by a variety of mechanisms. The elevated ERV activation detected in our studies may be a simple consequence of prion infection because pathological changes in prion disease, such as neuronal cell death, astrogliosis and changes in cytokine levels, are known to influence the expression and release of ERVs. However, the potential roles for transcribed ERV genes or their functional proteins in the pathogenic conditions of prion diseases cannot be excluded. Although further investigation is necessary to evaluate the precise threat posed by ERVs that contribute to prion pathogenesis, our findings combined with previous data suggest that ERVs appear to be closely associated with prion diseases.
